# Cardiovascular Disease Detection using Ensemble Learning

**DOI:** 10.1155/2022/5267498

**Published:** 2022-08-16

**Authors:** Abdullah Alqahtani, Shtwai Alsubai, Mohemmed Sha, Lucia Vilcekova, Talha Javed

**Affiliations:** ^1^College of Computer Engineering and Sciences, Prince Sattam Bin Abdulaziz University, AlKharj, Saudi Arabia; ^2^Faculty of Management, Comenius University in Bratislava, Odbojárov 10, 82005 Bratislava 25, Slovakia; ^3^Department of Computer Science, COMSATS University, Sahiwal, Pakistan

## Abstract

One of the most challenging tasks for clinicians is detecting symptoms of cardiovascular disease as earlier as possible. Many individuals worldwide die each year from cardiovascular disease. Since heart disease is a major concern, it must be dealt with timely. Multiple variables affecting health, such as excessive blood pressure, elevated cholesterol, an irregular pulse rate, and many more, make it challenging to diagnose cardiac disease. Thus, artificial intelligence can be useful in identifying and treating diseases early on. This paper proposes an ensemble-based approach that uses machine learning (ML) and deep learning (DL) models to predict a person's likelihood of developing cardiovascular disease. We employ six classification algorithms to predict cardiovascular disease. Models are trained using a publicly available dataset of cardiovascular disease cases. We use random forest (RF) to extract important cardiovascular disease features. The experiment results demonstrate that the ML ensemble model achieves the best disease prediction accuracy of 88.70%.

## 1. Introduction

The human heart is the body's most important organ, responsible for pumping blood to every body region. The circulatory system comprises arteries, veins, and capillaries, all of which connect to the heart [1]. The heart is one of the body's most essential organs, yet it is vulnerable to illness and injury. Because of its danger to human life and the illnesses and injuries it causes, its presence cannot be ignored. Heart disease affects the heart's pumping systems, causing it to malfunction [2]. Cardiovascular disease is a category of ailments that are caused by heart problems. Cardiovascular disease may be diagnosed by shortness of breath, physical weakness, swollen feet, and exhaustion. High cholesterol, smoking, sedentary lifestyles, and high blood pressure are a few risk factors for cardiovascular disease [3]. The leading cause of mortality, according to the World Health Organization [4], is cardiovascular disease, which kills 18 million people a year, or about 32% of all fatalities. The most common kind of heart disease is coronary artery disease. As a result, heart disease and stroke are major public health concerns.

Clinicians often employ angiography to diagnose cardiovascular disease. In underdeveloped nations, where diagnostic technologies, physicians, and other resources are few, this diagnosis process is time-consuming and costly since it requires examining many variables. Heart disease has become one of the most critical medical topics in recent years as the mortality toll from the cardiovascular disease continues to climb. Prediction aids in the early detection of disease and the most effective treatment. The use of ML in medical diagnostics is proliferating. This may be partly ascribed to improving the categorization and identification of illnesses, giving data to help medical professionals discover and diagnose diseases, sustaining human health, and decreasing the mortality rate. When determining the likelihood of a disease incidence, ML classification approaches are often used [5, 6]. This work's goal is a classification model that can predict cardiovascular disease.

Making predictions based on experience has become a central goal of ML research. The neural network is one of the most common methods of machine learning. This is the first step in supervised learning, in which a model is built using labeled data, and its performance is assessed using test data. Classification and regression difficulties are common in supervised learning. This is the second form of learning, where the data is not labeled, and the model attempts to uncover the hidden patterns in the data. Data exploration results generate inferences about the nature of any concealed knowledge. Unsupervised learning is shown by the clustering method [7]. Reinforcement learning is the third form, which does not employ labeled data and does not link the conclusions to the data. An environment's intelligent agents [8] focus on this research. Use a real-world dataset of patients with cardiovascular illness to construct a classification model. This ML method is often used to predict the likelihood of illness occurrence [5, 6]. To forecast the class of fresh samples, a model learned from training data is used [9, 10]. Categorizing data into classes is also a supervised learning notion [10].

This work provides contributions to detecting cardiovascular disease more effectively and efficiently. Below are the major contributions of this paper.

- Proposed ensemble learning method for detecting cardiovascular disease including several classifiers in voting-based decision-making.
- Compare the proposed method to traditional machine learning approaches such as RF, K-nearest neighbor (KNN), multi-layer perceptron (MLP), and State-of-the-art research to determine the efficacy of the proposed approach.
- Experimental results show a 1.47% accuracy improvement compared to earlier state-of-the-art research and standard machine learning algorithms for the proposed approach.

Section 2 provides an overview of the relevant literature. Details regarding the proposed approach and the dataset are provided in section 3, and the outcomes and comparisons are provided in Section 4 in the comparative part. Finally, Section 5 concludes this work and provides a detailed description of future research.

## 2. Literature Review

Machine learning has sparked widespread interest in various fields, including health and medicine [11]. Researchers have proposed numerous viable approaches to detecting cardiac disease using machine learning. Consequently, studies on developing medical applications employing various machine learning algorithms and approaches have been published.

According to [12], machine learning may be used to measure and predict people's cognitive health using intelligent home sensors that have been built. A total of 179 individuals were viewed as volunteers by the respondents. We found an *r* = 0.79 association between the automated task quality evaluation ratings and the direct observation scores. Participants' cognitive health was predicted using machine learning approaches with an AUC of 0.64.

According to [13], machine learning may be used to detect cardiovascular illness based on a patient's clinical data. The RF model was the most accurate, with an accuracy rate of 86.60%. Motarwar et al. [14] developed an algorithmic system to predict heart disease risk. Their findings, at 95.08%, discovered that RF was the most accurate. Multiple algorithms, including Decision Tree (DT), NB, KNN, and RF, were used by Sabarish and Parvati [5]. According to the data, the KNN algorithm has the highest accuracy rate (90.7%). Additionally, a CNN model was used to detect images of Chinese herbal medication, achieving a 71% total accuracy. With an incredible 96.7% success rate, the usage of ANN for lung cancer detection was also found [15].

Some studies look at ways to improve the status quo and develop new classifiers. Using Natural Language Processing (NLP) to train and assess a depression prediction model, for example, was proposed in research by [16]. According to [17], a neural network was proposed for predicting diabetes, with a prediction accuracy of 87.3%. Compared to its baseline performance of 89% and 81%, the proposed approach enhanced the neural network's performance by 91% in the training and 86% in the testing.

It was hypothesized in [18] that the Particle Swarm Optimization (PSO) approach might be used to enhance a neural network's accuracy further. The research was based on a dataset that included 303 healthy and ill people cases. There were 72 features in the dataset, but only 13 of them were employed in the feature selection process using PSO. The PSO was utilized for feature selection and ranking after preprocessing the dataset. From a pool of thirteen options, ranking results indicated the eight most valuable options for improving neural network training accuracy using a feedforward backpropagation approach. According to the study's findings, the hybrid PSO-FFBP neural network was 91.94% accurate in predicting heart illness. It was also shown that diabetic retinopathy might be predicted using an upgraded version of the DNN using the Grey Wolf Optimization (GWO) algorithm and PCA. A questionnaire was developed by Nowshad et al. [19] in the Sylhet area of Bangladesh, where they visited many local hospitals and health care facilities to collect data. Their dataset has 564 occurrences and 18 characteristics. The SVM was the most accurate, with a 91% success rate.

Using machine learning approaches to identify key traits, Mohan et al. [20] have created a method for improving cardiovascular disease prediction accuracy. The proposed mixed RF and linear model method were 88.7% accurate in predicting heart disease. Au et al. [21] have presented a hybrid approach for diagnosing cardiac disease. Logistic regression was shown to have an accuracy rate of 89% when predicting heart disease. Authors in [22] proposed a hybrid model for predicting coronary artery disease. They used three machine learning algorithms: RF, DT, and a mix of the two to create the model. The hybrid model was the most accurate, with an accuracy of 88.7%.

According to [23], they used standalone machine learning approaches to detect heart disease. They used different ensemble learning models to improve the accuracy of a prediction model. They used sleep disorder, stress mismanagement, and pollution factors as features.

For this study, we utilize data on cardiovascular disease. This dataset has around 70000 patients and 11 characteristics, which we believe is the third research to use. Also, we use various machine learning algorithms and deep learning algorithms to find the best one for predicting cardiovascular disease.

## 3. Proposed Approach

**Figure 1:**
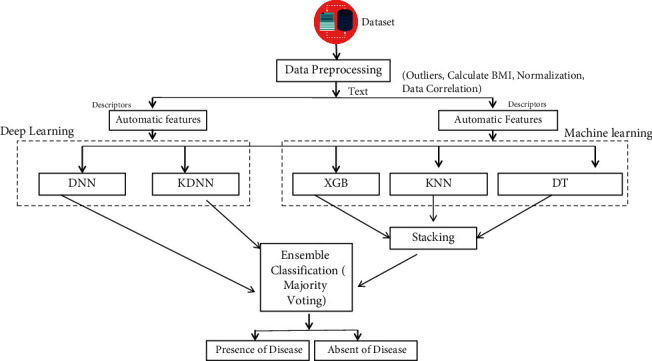
A proposed approach for cardiovascular disease detection.

This section describes the methods to predict cardiovascular disease.  Figure 1 depicts the proposed approach comprising six phases. The proposed approach begins with the selection of a dataset for the experiment. The Cardiovascular Disease dataset is used for experiments in this research. The preprocessing stage includes many steps that must be completed before model training. The features extraction approach is then utilized to determine the relevance of the features, and finally, several machine learning and deep learning classifiers are employed for experiments. Deep learning methods are also being evaluated in this study to detect cardiovascular disease. To identify the existence of cardiovascular disease, four prior ML classifiers are used: RF, KNN, DT, and Extreme Gradient Boosting (XGB). The ability of deep learning models on a particular dataset is tested using two deep neural network classifiers.

### 3.1. Dataset Selection

The Kaggle repository's Cardiovascular Disease dataset (https://www.kaggle.com/datasets/sulianova/cardiovascular-disease-dataset) is utilized in this research. There are 70,000 samples in the dataset and 13 attributes. 70% data is utilized for training models, while the remaining 30% is used to assess the model's performance.  Table 1 shows the description of the features and explains each feature in-depth.

### 3.2. Data Preprocessing and Corelation Analysis

Machine learning relies on data preprocessing to assess data quality and retrieves critical information that can influence the learning model's performance. Before training a model, preprocessing is essential. During the preprocessing stage, addressing diverse dataset characteristics, handling missing values, scaling, and standardization are dealt with during the preprocessing stage. This includes managing small, huge, and noisy datasets, overcoming overfitting, dealing with class imbalance, and label encoding. This work recommends using preprocessing approaches such as eliminating anomalies (outliers) and applying a standard scaler to the dataset to demonstrate the model's efficiency and acquire an acceptable and dependable accuracy for disease prediction. We employ a standard scaler to normalize the data within a predetermined range. It operates by altering characteristics like its distribution to reach a mean of 0 and a standard deviation of 1. Equation (1) defines a standard scaler, where *S* is the standardized form of *z*_*i*_.

(1) $$\begin{array}{c} S=\frac{\left(z_{i}-\mu \right)}{\sigma }. \end{array}$$

ML models make better decisions depending on how labels are used. The Label Encoder transforms the target labels into numeric forms so that machines can read them for improved disease diagnosis. We also made the following changes to the dataset to identify better the characteristics that most affect cardiovascular disease:

- Body Mass Index (BMI) combines weight and height into a single characteristic that evaluates body fat percentage depending on height and weight.
- To make the dataset more understandable, features are modified while the information is preserved. The gender feature is converted to binary, and the age is translated from days to years in this dataset.
- Drop the dataset's year, height, weight, and id columns.
- Checking the maximum and minimum of quartiles to identify outliers and remove the rows that have outliers.

**Figure 2:**
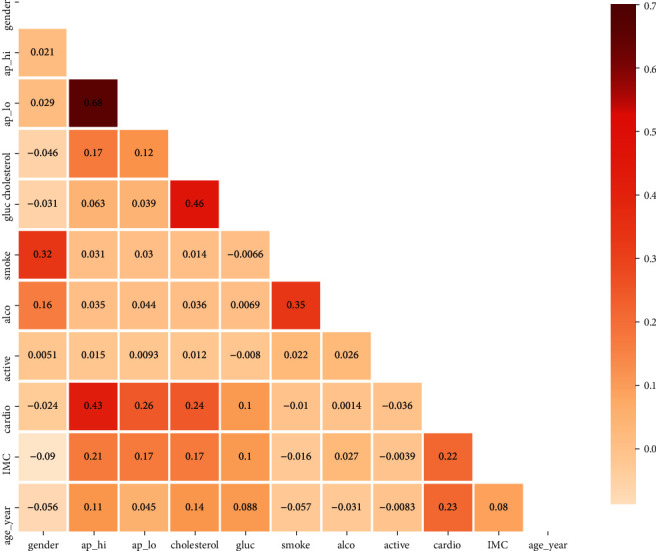
Corelation feature matrix.

Pearson's coefficient (PCC) is used to get the corelationship between the characteristics to remove nonessential, duplicate, and redundant features from the data, as shown in Figure 2. The correlation coefficient might be anywhere from −1 to 1. The features are negatively correlated if the value is close to −1; if the value is close to 1, the features are tightly coupled and considerably influence model performance. We set the threshold values to 0.85% to determine the PCC; if the correlation value is above the threshold, the feature is ignored; if the correlation value is below the threshold, the feature is kept. All characteristics are the same after the feature corelation analysis.

### 3.3. Feature Extraction

It is necessary to select essential features that aid in classification. Its goal is to minimize dimensionality by identifying the most informative feature that may help the models perform better. We found that the RF feature selection approach identifies the essential features. It is also a prominent feature selection approach in machine learning methods, and it is worked well in real-world scenarios.

### 3.4. Classification Models

We propose a machine learning-based ensemble model (XGB, KNN, and DT) and an ML and DL-based stacked model (ML ensemble model, DNN, and KDNN) to improve the classification results. We applied four machine learning methodologies for our experiment: RF, KNN, DT, XGB, and two deep learning models, DNN and KDNN. This section shows how to use the machine learning ensemble classifier and DL method to identify cardiovascular disease and evaluate our approach's success effectively.

### 3.5. Machine Learning Ensemble Classifier

Researchers have used ensemble learning to solve a range of machine learning problems [24, 25]. Because each dataset contains a wide range of problems, an ensemble learning approach is used to distinguish different diseases and categorize data appropriately. Every classifier in the ensemble estimates a class label whenever a new data point comes in. The class label that is predicted by the majority of the classifiers, or the class label with the most votes, is the one that is used for that instance. Try to improve performance by using a mix of ML-classifiers and different ways to vote [26]. These strategies are better than the classic single-learning approach for better generalization results.

The proposed ML ensemble classifier takes the predictions of several classifiers and uses a weighted majority method to develop the results. After each classification model has been fine-tuned, the best results are reported. The method in the equation is used to get the most votes in equation (2).

(2) $$\begin{array}{c} \overset{\approx }{Z}=\arg\max\left(X_{1}\left(Z_{i}^{1}\right),X_{2}\left(z_{i}^{2}\right),\ldots ,X_{i}\left(z_{i}^{n}\right)\right). \end{array}$$

K-Nearest Neighbour (KNN) is the essential machine learning approach for regression and classification. The data is used in KNN computations, which utilize similarity measurements (e.g., distance function) to describe new points. The KNN calculation relies on the similarity of the items being compared. In KNN, classification is determined by the majority vote of its immediate neighbors. The class with the most neighbors is labeled on the data point [27]. As the number of nearest neighbors increases, the choice of *k* and the accuracy may improve. We used the default parameter settings given by the sklearn library.
Extreme Gradient Boosting helps to improve performance and use of memory resources. This ensemble learning approach uses XGBoost to improve the performance of classification. The XGBoost classifier is used to improve the accuracy of classification. XGBoost is a machine learning algorithm used on a large-scale dataset. It uses gradient descent to optimize the loss function and returns a prediction as a boosting ensemble of weak classification trees [28]. This model is unique in combining weak ML algorithms with an agile approach to learning. Gradient boosting is one of the few models of its sort. Gradient boosting uses the residual error to change the previous prediction to optimize the loss function. With XGBoost, the loss function is regularized to offer an objective function for assessing the model's performance, which is defined by
  (3) $$\begin{array}{c} X^{\prime }\left(⊕\right)=\text{Loss}\left(⊕\right)+\Omega^{\prime }\left(⊕\right). \end{array}$$
Equation (3) indicates the parameters that may be inferred from the available data by adding oplus. The model's complexity is measured by the loss function (Loss) and the regularization term (Omega′). The XGBoost algorithm parameters utilized in this research are as follows: The maximum depth parameter is used by the XGBoost model. 6 is the highest depth that may be reached. Booster is gbtree, eta value is 0.3 in addition to the following parameters: scale position weight is 1, min-children-weight is also 1, and finally, the booster is 1 and all other parameters remain default.
Decision Tree may be used to sort categorical and numerical data alike. It resembles a tree in terms of structure. When working with medical data, DT is the most popular approach. Creating and analyzing a tree-shaped graph is a cinch. As one of the most effective and extensively utilized methods for regulating learning, we turned to the DT classification method [29]. Building a stable decision tree for a specific data collection is straightforward. This model also used the default parameters while training on the given dataset.

### 3.6. Deep Learning Classifier

Deep neural networks have been used as DL-based classifiers (DNN). Recurrent neural network (RNN) models such as LSTM have also been employed, although LSTM did not perform well on the given dataset because of the abundance of numeric input. We used DL-based classifiers to evaluate cardiovascular disease detection and classification performance to provide a thorough study. The input layer with a ReLU activation function of 10 dimensions and a unit value of 16 is followed by three dense layers, each with a unit value of 12, 8, and 4, and a fully-connected layer with a sigmoid activation function associated directly with a specific value that the model is trying to predict; this model is called the DNN model. A total of 176 parameters make up the input layer. This layer has 204 parameters, each with an individual value of 12. One hundred four parameters with eight units are set in the second hidden layer, and 36 parameters with four in the final hidden layer. The learning rate of the DNN model is 0.001, and the loss is calculated using Binary cross-entropy in the DNN model.

It is possible to learn all the parameters, and the proposed model performed well on the test data. To prevent overfitting, we used a neural network known as KDNN, a robust medical disease detection approach. When we reduced the size of the dataset by removing outliers, we applied an effective medical disease detection Neural Network (KDNN) to control overfitting. The Keras Deep Neural Network (KDNN) model employs an Adam optimizer with Binary cross-entropy to compute the loss and simplify fine-tuning. The proposed KDNN model has a single input layer as its architecture. Finally, a single, fully connected layer is revealed after many hidden layers. Finally, a single, fully connected layer is revealed after many hidden layers. The 132 parameters are presented in the input layer. One hundred thirty parameters are buried in the following layer. The third hidden layer has 88 parameters, while the final hidden layer has 54 parameters with 411 training parameters. This fine-tuned KDDN model is successfully trained and tested on the Cardiovascular Disease dataset.

## 4. Results and Discussion

This section briefly describes the results and compares the baseline methods. This study's major objective is to examine the classification performance of recommended machine learning and deep learning classifiers for cardiovascular disease detection. The experiments for this study are done with the Cardiovascular Disease dataset. Both machine learning and deep learning are used in this study. The experiments are done in two different ways. Machine learning models are used in the first step to getting better results. We devised an ML-based ensemble model that performed well on the given dataset. This is done to get more reliable results. In the second step, we conducted experiments with deep learning approaches. Because there is not much data to work with, the deep learning models did not do well, so we made a stacked ensemble model to get better results from deep learning. This study used several measures of performance: accuracy, precision, recall, F1 score, confusion matrix, and area under the ROC curve (AUC-ROC score). The dataset is split into two parts: 70% of the data is used to train the model, and 30% is used to test the model. Python 3 and a Jupyter notebook running on an i5-8300H laptop with 16 GB RAM and a GTX 1050 2 GB VRAM are used to simulate the proposed model.

### 4.1. Experimental Results

**Figure 3:**
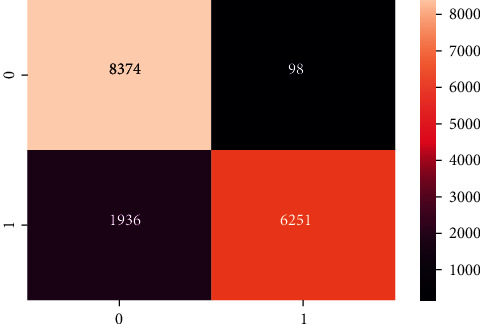
Confusion matrix of random forest classifier.

**Figure 4:**
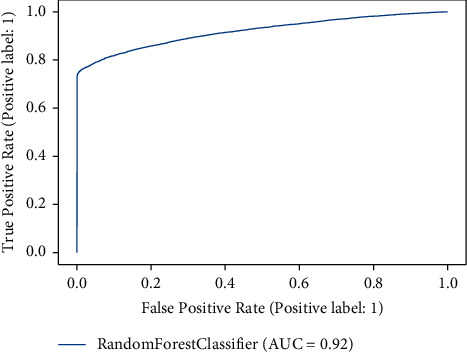
ROC AUC curve of random forest classifier.

Table 2 demonstrates the results of the machine learning classifiers on the Cardiovascular Disease dataset to identify the existence of cardiovascular disease. RF, KNN, DT, and XGB are employed in this research. Table 2 demonstrates that, compared to other machine learning models, the RF classifier obtained the best accuracy rate of 88.65%. RF's precision, recall, F1, and AUC scores are 90.03%, 80.03%, 80.02%, and 92%, respectively. We also plot the confusion matrix of the RF classifier.  Figure 3 shows the confusion matrix of the RF classifier, where diagonal values are accurately classified by the RF classifier and nondiagonal are misclassified by the RF classifier. In addition, Figure 4 shows the ROC Curve plot with the ROC score of 92%, representing that the RF classifier performed very well on the Cardiovascular Disease dataset.

**Figure 5:**
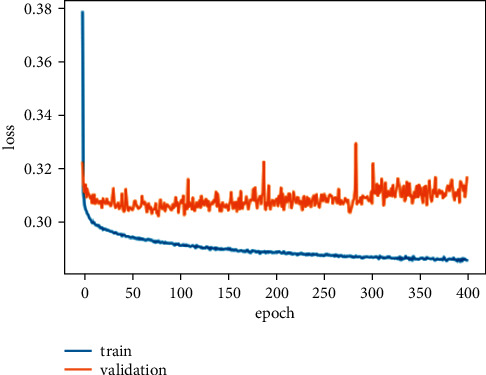
Training and validation loss of DNN model.

**Figure 6:**
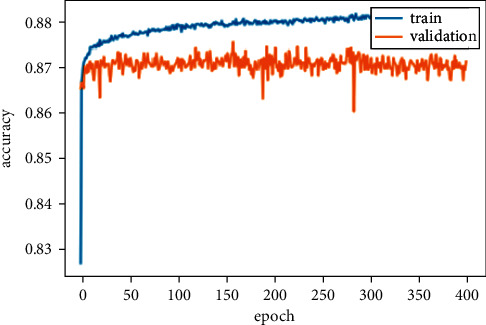
Training and validation accuracy of DNN model.

The second stage of the experiments shows the deep learning model results. The deep learning model results are presented in Table 3. Both the deep learning models performed exceptionally well. Both models' accuracy score is almost the same but less than the machine learning model because the deep learning model performed well on a large amount of data. The DNN model gets the highest accuracy than the KDNN model. The accuracy, precision, recall, F1 score, and ROC AUC score of the DNN model are 87.59%, 97.77%, 76.27%, 65.54%, and 91.50%. We also computed the loss of the DNN model, which is 30.19%. The training and validation loss of the DNN model is shown in Figure 5 while Figure 6 shows the training and validation accuracy of the DNN model on 400 epochs.

**Figure 7:**
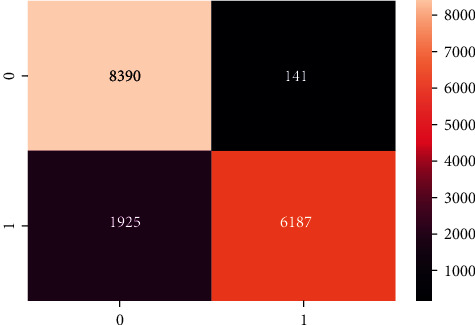
Confusion matrix of DNN model.

**Figure 8:**
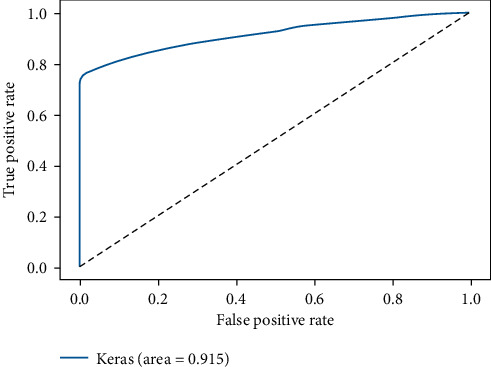
ROC AUC curve of DNN model.

The confusion matrix of the DNN model is presented in Figure 7, and the ROC AUC curve plot is shown in Figure 8.

**Figure 9:**
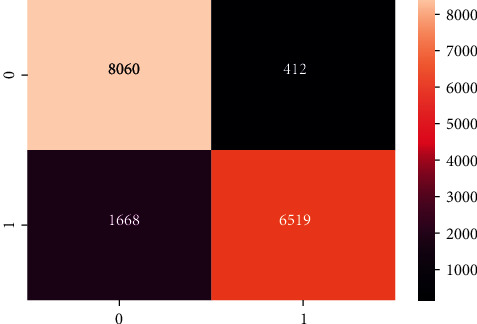
Confusion matrix of ML Ensemble model.

**Figure 10:**
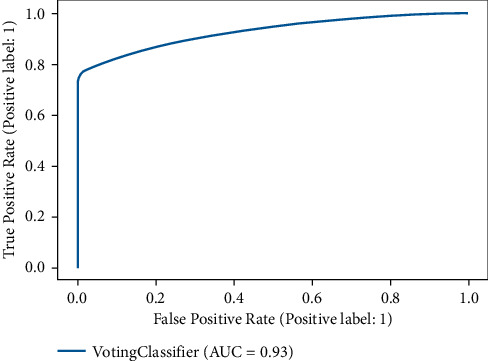
ROC AUC curve of ML ensemble model.

A machine learning ensemble classifier and an ML and DL stacked classifier are used to get the final findings in this study. This classifier has the greatest accuracy of 88.70% among all other classifiers. However, the lack of data meant that the stacked classifier did not perform effectively. ML Ensemble has an 88.02% precision and recall rate, an 88.01% F1 score, and a 93% ROC AUC. The confusion matrix and AUC ROC of the proposed ML Ensemble classifier are shown in Figures 9 and 10. The results show that the proposed ML ensemble classifier can efficiently detect the presence of cardiovascular disease from the Cardiovascular Disease dataset.

Table 4 provides the results for the ensemble ML model consisting of ML and DL stacked classifiers to detect cardiovascular disease. The stacked classifier achieved the accuracy of 86.49%, Precision of 87.32%, Recall of 86.02%, F1 score of 86.01%, and ROC-ACU of 99% while the ML ensemble model achieved the accuracy of 88.70%, Precision of 88.02%, Recall of 88.02%, F1 score of 88.01%, and ROC-ACU of 99%. To sum up, the ML model achieved the best results in detecting cardiovascular disease.

### 4.2. Comparative Analysis

The comparative analysis of the proposed and baseline approaches is presented in Table 5. We broadly compare with two baseline research studies [30, 31]. The experimental setting of the proposed and baseline studies are almost the same. This research used an ML Ensemble method to detect cardiovascular disease.

In Table 5, we compare this study's findings to our baseline method. Alfaidi et al. used various ML models (LR, RF, DT, NB, KNN, SVM, and MLP) and achieved the highest result from the MLP model with 87.23% accuracy [30]. Arroyo and Delima used two Artificial neural networks, and five ML models (LR, DT, RF, SVM, and KNN) [31]. This study obtained the highest accuracy using the GA-ANN model, 73.43%. The proposed approach used a majority voting approach and created an ML Ensemble model, which obtained the highest accuracy compared with the baseline results. The ML Ensemble model obtained an accuracy score of 88.70%. The accuracy gain of 1.47% using the ML Ensemble model shows that the proposed ML Ensemble performed efficiently to detect cardiovascular disease.

## 5. Conclusion

This study proposed ensemble-based machine and deep learning approaches to predict cardiovascular disease. For this study, we used 70000 patients' data with various forms of cardiovascular disease. The accuracy of the models was used as a measure of their performance. In addition, we selected additional informative characteristics that influence the models' performance. According to the results, the ML Ensemble model was the most accurate in predicting cardiovascular disease. We have added a few procedures to get the dataset ready for analysis. We may employ various strategies to find the best features for our future work. For a better and more accurate assessment, additional datasets may be employed. Finally, deep learning and reinforcement learning approaches may tackle the prediction issue to detect cardiovascular disease more efficiently.

## Figures and Tables

**Table 1:** Dataset features description.

No	Feature name	Type	Description
1	ID	Integer	Id number
2	Age	Integer	Number in days
3	Gender	Integer	Men: 2, women: 1
4	Height	Integer	In cm, max = 250, min = 55
5	Weight	Continuous	In kg, max = 200, min = 10
6	Systolic blood pressure	Integer	Max = 16020, min = −150
7	Diastolic blood pressure	Integer	Max = 11000, min = −70
8	Cholesterol	Integer	(1) Normal, (2) above normal, (3) well above normal
9	Glucose	Integer	(1) Normal, (2) above normal, (3) well above normal
10	Smoking	Integer	Binary
11	Alcohol intake	Integer	Binary
12	Physical activity	Integer	Binary
13	Cardiovascular disease	Integer	Binary

**Table 2:** Machine learning classifier performance (%) for cardiovascular disease detection.

Model	Accuracy	Precision	Recall	F1 score	ROC-AUC
RF	88.65	90.03	88.03	88.02	92
KNN	86.45	87.53	86.21	86.25	90
DT	86.35	86.23	86.22	86.22	88
XGB	88.19	88.25	88.01	88.01	94

**Table 3:** Deep learning classifier performance (%) for cardiovascular disease detection.

Model	Accuracy	Precision	Recall	F1 score	ROC-AUC
DNN	87.59	97.77	76.27	65.54	91.50
KDNN	87.54	98.52	75.57	65.54	91.49

**Table 4:** ML ensemble and DL stacked classifier performance (%) to detect cardiovascular disease.

Model	Accuracy	Precision	Recall	F1 score	ROC-AUC
ML ensemble	88.70	88.02	88.02	88.01	93
Stacked classifier	86.49	87.32	86.02	86.01	99

**Table 5:** Comparison of proposed and baseline approaches classifiers performance to detect cardiovascular disease.

Method	Models	Accuracy (%)
Baseline approach [30]	LR	85.54
	RF	86.03
	DT	85.93
	NB	83.38
	KNN	84.56
	SVM	86.63
	MLP	87.23

Baseline approach [31]	GA-ANN	73.43
	ANN	68.35
	Logistic regression	72.35
	Decision tree	61.72
	Random forest	68.94
	Support vector machine	72.16
	K-nearest neighbor	68.34

Proposed approach	ML ensemble	88.70

## Data Availability

The Cardiovascular Disease data used to support the findings of this study are included in the article.
